# Low-Dose Ribavirin Treatments Attenuate Neuroinflammatory Activation of BV-2 Cells by Interfering with Inducible Nitric Oxide Synthase

**DOI:** 10.1155/2015/923614

**Published:** 2015-08-27

**Authors:** Iva Bozic, Danijela Savic, Marija Jovanovic, Ivana Bjelobaba, Danijela Laketa, Nadezda Nedeljkovic, Mirjana Stojiljkovic, Sanja Pekovic, Irena Lavrnja

**Affiliations:** ^1^Department of Neurobiology, Institute for Biological Research “Sinisa Stankovic”, University of Belgrade, Boulevard Despot Stefan 142, Belgrade 11060, Serbia; ^2^Institute of Physiology and Biochemistry, Faculty of Biology, University of Belgrade, Studentski Trg 3, Belgrade 11001, Serbia

## Abstract

Microglia play a key role in defending central nervous system from various internal and external threats. However, their excessive and/or chronic activation is associated with deleterious effects in a variety of neurodegenerative diseases. Previously, we have shown that ribavirin when applied in clinically relevant dosage (10 *μ*M) modulates activated microglia in complex fashion inducing both anti- and proinflammatory effects, simultaneously causing cytotoxicity. Here, we examined potential of low-dose ribavirin (0.1 and 1 *μ*M) to modulate activated BV-2 microglia. Morphological and functional activation of BV-2 cells was achieved with lipopolysaccharide (LPS) stimulation. Our results demonstrated that low-dose ribavirin did not induce cell death, while 10 *μ*M ribavirin promoted LPS induced apoptosis. We determined that 1 *μ*M ribavirin was equally efficient in deactivation of LPS induced morphological changes as 10 *μ*M ribavirin treatment. Ribavirin showed halfway success in reducing markers of functional activation of microglia. Namely, none of the doses had effect on LPS triggered production of proinflammatory cytokine tumor necrosis factor alpha. On the other hand, low-dose ribavirin proved its effectiveness in reduction of another inflammatory mediator, nitric oxide, by inhibiting inducible form of nitric oxide synthase. Our results imply that low-dose ribavirin may alleviate nitrosative stress during neuroinflammation.

## 1. Introduction

Microglia are resident cells of the central nervous system (CNS) with immune function. Once they sense pathogen- or danger-associated molecular patterns, microglia cells go through morphological and functional activation [1]. Activated microglia migrate to the endangered area, exert phagocytic activity to remove external antigen and potential deleterious debris, and begin to secrete number of proinflammatory factors, such as tumor necrosis factor-alpha (TNF-*α*), nitric oxide (NO), reactive oxygen species, and prostaglandin E2 [2]. In this way tissue repair is promoted, so it is generally accepted that activation of microglia is beneficial [3–5]. On the other hand, excessive microgliosis or sustained chronic activation of microglia underlies many neurological disorders, including multiple sclerosis [6].

Ribavirin (RBV, 1-*β*-D-ribofuranosyl-1,2,4-triazole-3-carboxamide, also known as Virazole) is a synthetic guanosine analogue exerting strong antiviral activity against variety of RNA and DNA viruses [7]. Biochemical and pharmacological data revealed that primary molecular target of ribavirin action is inosine monophosphate dehydrogenase (IMPDH), a key enzyme in* de novo* synthesis of guanine nucleotides [8]. Inhibition of IMPDH by RBV results in cellular depletion of GTP. Apart from viruses, different cell types of immune system, including subtypes of T cells [9–12], macrophages [13], and dendritic cells [14], are sensitive to RBV action. Immunomodulatory and immunosuppressive actions of RBV have been additionally evidenced in experimental autoimmune encephalomyelitis (EAE), an* in vivo* animal model of multiple sclerosis. RBV strongly affects immune branch of EAE as evidenced by decreased number of mononuclear cell infiltrates [15] and suppressed production of proinflammatory cytokines IFN-*γ*, IL-1*β*, and TNF-*α* in draining lymph nodes [16]. As result of prevented infiltration of the immune cells in the CNS during EAE, RBV modulated glial cell response, indicated by smaller number of reactive astrocytes [17] and activated microglial cells [15]. It is of note that RBV readily crosses blood-brain barrier [18, 19], especially the one compromised by neuroinflammation [20]. Therefore, RBV might directly act on glial cells within CNS. Indeed, we have shown that RBV has capability to modulate activated microglia* in vitro* [21]. However, dosage of ribavirin (10 *μ*M) applied on activated primary microglia, although therapeutically recommended [22], induced anti- and proinflammatory properties with simultaneous moderate cytotoxicity [21].

Therefore, in the present study we used BV-2 microglial cell line activated with bacterial wall lipopolysaccharide (LPS) to explore the potency of RBV when applied in low-doses, which are ten to hundred times lower than clinically relevant ones. In such experimental design, we have evaluated the ability of RBV to induce apoptosis, alterations in cell morphology, release of TNF-*α*, NO production, and induction of inducible nitric oxide synthase (iNOS), which constitute the hallmarks of activated microglia* in vivo*.

## 2. Material and Methods

### 2.1. Cell Culture

BV-2 microglial cell line was a generous gift from Dr. Alba Minelli (University of Perugia, Perugia, Italy). Cells were cultured in RPMI 1640 medium supplemented with 10% heat inactivated fetal calf serum (FCS) and 1% penicillin/streptomycin, all purchased from PAA Laboratories GmbH, Pasching, Austria. When cultures reached confluence, they underwent passages by trypsinization and were seeded in different plates depending on the experiment.

The cells were pretreated with RBV (0.1, 1, and 10 *μ*M) for 30 minutes prior to stimulation with LPS (100 ng/mL) from* Escherichia coli* serotype 026:B6 (Sigma-Aldrich Chemie GmbH, Munich, Germany) for additional 24 h. The treatment protocol was applied in all experiments of this study. RBV was a kind gift from MP Biomedicals, LLC (Illkirch, France).

### 2.2. Flow Cytometry

BV-2 cells (2.5 × 10^5^/well) were seeded in 6-well plates, treated with ribavirin and LPS as described above. Assessment of cell viability involved double staining of cells with Annexin V-FITC (Santa Cruz, Dallas, Texas, USA) and propidium iodide (PI; BD Pharmingen, San Diego, CA, USA). Annexin V binds to phosphatidylserine, exposed on the surface of early apoptotic cells, while PI uptake is marker for necrotic or later apoptotic cell death. Negative staining for both dyes was characteristic of viable cells. Staining was performed according to the manufacturer's instructions. Flow cytometry was conducted on CyFlow Space Partec (Partec GmbH, Munster, Germany) and the data was analyzed using PartecFloMax software (Partec GmbH, Munster, Germany).

### 2.3. Morphological Analysis

Morphological analysis was performed using phalloidin fluorescence microscopy. Cells were plated at 8 × 10^4^ on glass coverslips (Ø25 mm) in 35 mm dishes (Sarstedt, Newton, NC, USA). After the treatment, cells were fixed with 4% paraformaldehyde for 20 min at 4°C, washed with PBS, and then permeabilized with Triton X-100 (0.25%, Sigma-Aldrich, Munich, Germany) for 15 min. After blockade in 5% bovine serum albumin (BSA, Sigma-Aldrich, Munich, Germany) actin filaments were stained by incubating cells (30 min, RT) with Alexa Fluor 555 phalloidin (Invitrogen, Carlsbad, CA, USA) at 1 : 50 in PBS. Cells were washed with PBS and counterstained with Hoechst 33342 (5 *μ*g/mL, Life Technologies, Invitrogen, Carlsbad, CA, USA). Cells were coverslipped with Mowiol (Calbiochem, Darmstadt, Germany) and images were acquired using Zeiss Axiovert fluorescent microscope (Zeiss, Jena, Germany). To quantitatively characterize cell morphology we used AxioVision Rel. 4.6 software, which automatically measures the 2D cell surface area. Cells were analyzed in five random areas (138 × 104 *μ*m^2^) per coverslip, with three coverslips for each group, in three independent cell preparations.

### 2.4. NO Production

Concentrations of NO in the culture supernatant were determined by measuring nitrite, a major stable product of NO, using the Griess reagent. Cells (5 × 10^4^/well) were cultured in 24-well plates and treated as described. Aliquot of 100 *μ*L of each culture medium was mixed with an equal volume of Griess reagent (1% sulfanilamide (Sigma, Munich, Germany)/0.1% N-(1-naphthyl)-ethylenediamine dihydrochloride (Fluka, Buchs, Switzerland)/2.5% H_3_PO_4_). Spectrophotometric measurements were performed using the LKB 5060-006 ELISA plate reader, at 540/670 nm test/reference wavelengths. Nitrite concentrations were calculated from the standard curve generated using known concentrations of sodium nitrite (NaNO_2_; Merck, Darmstadt, Germany).

### 2.5. Western Blot Analysis and Immunofluorescence

BV-2 cells were plated in 6-well plates at a density of 2.5 × 10^5^ cells/well, treated for 24 hours, harvested by trypsinization, and centrifuged at 750 ×g for 3 min. Pelleted cells were lysed in ice-cold lysis Triton X-100 buffer (50 mM Tris-HCl, pH 7.4, 150 mM NaCl, 1% Triton X-100, and 0.1% sodium dodecyl sulphate (SDS)) in which protease inhibitor (Roche, Penzberg, Germany) was added. Cell lysates were centrifuged at 17 900 ×g for 20 min at 4°C, and supernatants were collected. Protein content was determined using the BCA protein assay kit (Pierce Biotechnology, Rockford, IL, USA). Protein samples (20 *μ*g) were separated by 7.5% SDS polyacrylamide gel electrophoresis with 100–120 V and transferred onto a polyvinylidene fluoride (PVDF) membrane (Roche, Penzberg, Germany) for 1 h at 100 V with cooling. The membrane was blocked with 5% BSA dissolved in Tris-buffered saline Tween-20 (TBST) (20 mM Tris, pH 7.6, 136 mM NaCl, and 0.1% Tween-20) for 1 h at room temperature. Membranes were incubated with anti-iNOS antibody (1 : 500; Abcam, Cambridge, UK) overnight at 4°C, washed with TBST for 10 min three times, and then incubated with HRP-conjugated secondary antibody (1 : 5000; Santa Cruz, Dallas, Texas, USA) for 1 h at room temperature. After washing, protein bands were visualized using chemiluminescence and developed onto film (KODAK). The relative expression levels of proteins were determined by densitometry using ImageQuant 5.2 software and were normalized against *β*-actin. The results are expressed as the percentage of control (nontreated cells). Data presented in graphs are mean values ± standard error of the mean obtained from four immunoblots.

For immunofluorescence labeling, cells (8 × 10^4^) were plated on glass coverslips (Ø25 mm) in 35 mm dishes. After 24 h treatment, cells were fixed, washed, permeabilized, and blocked with 5% BSA as described previously. Primary rabbit anti-iNOS antibodies (1 : 700; Abcam, Cambridge, UK) were applied overnight at 4°C. Cells were rinsed and incubated with fluorophore-labeled secondary antibodies (1 : 500; donkey anti-rabbit Alexa-488, Invitrogen, Carlsbad, CA, USA) for 1 h at room temperature. After rinsing the cells with PBS, nuclei were counterstained with Hoechst 33342 and coverslips were mounted with Mowiol. For the negative control of staining, the same procedure was applied without incubating the cells with the primary antibodies.

### 2.6. Enzyme-Linked Immunosorbent Assay

Enzyme-linked immunosorbent assay (ELISA) was used to determine the levels of TNF-*α* in cell-free supernatants. Cells were seeded in 24-well plates (5 × 10^4^/well), treated for 24 hours as described, and culture supernatants were collected. Levels of TNF-*α* were measured using the commercial kit (eBioscience, Frankfurt, Germany) according to the manufacturer's protocol. Briefly, after incubation with biotinylated detection antibody, avidin-HRP conjugate and subsequently chromogenic substrate 3,3′,5,5′-tetramethylbenzidine (TMB, eBioscience, Frankfurt, Germany) were added. Color development was ceased by adding 1 M H_3_PO_4_ and absorbance was measured at 450 nm. Concentrations of TNF-*α* in the culture medium were determined using the standard curve generated using known concentrations of recombinant murine TNF-*α*. Release values were calculated as pg cytokine per mL.

### 2.7. Statistical Analyses

Data values represent the mean ± SEM. Statistical significance was determined using analysis of variance that was followed by Bonferroni's test. A value of *P* < 0.05 was considered as statistically significant.

## 3. Results

### 3.1. Cell Viability Assay

**Figure 1:**
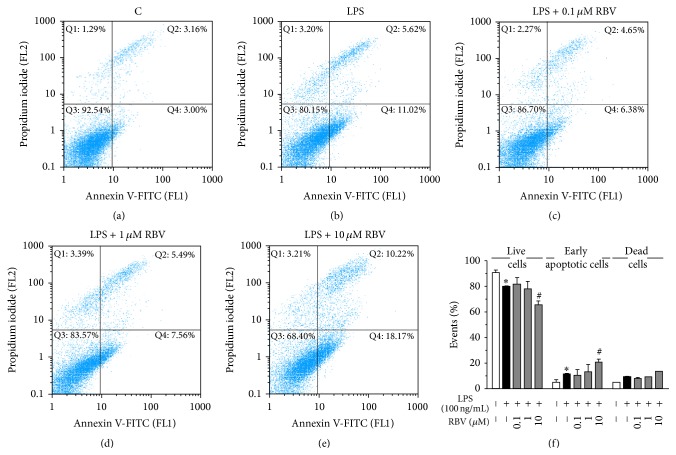
Annexin V/PI staining was analyzed with FACS after 24 h of RBV treatment and LPS stimulation. Representative dot plots are shown for control (a), LPS (b), and LPS stimulated BV-2 cells treated with 0.1 *μ*M (c), 1 *μ*M (d), and 10 *μ*M RBV (e). (f) Histogram representing percentage of live, early apoptotic, and dead cells after RBV pretreatment and LPS stimulation. Data represent mean ± SEM from three independent cell preparations. ^*∗*^ *P* < 0.05 versus control group, ^#^ *P* < 0.05 versus LPS stimulated group.

The effect of low-dose RBV treatment on viability of BV-2 cells (Figure 1(a)) in the culture was evaluated after 24 h, by Annexin V/Propidium iodide staining, which differentiates between live (Annexin V^−^/PI^−^), early apoptotic (Annexin V^+^/PI^−^), late apoptotic (Annexin V^+^/PI^+^), and necrotic cells (Annexin V^−^/PI^+^). LPS decreased the number of viable cells in culture by increasing the number of early apoptotic cells (Figures 1(b) and 1(f)). RBV administrated at the highest concentration (10 *μ*M) added to the effect of LPS, by promoting the apoptosis (Figures 1(e) and 1(f)). In contrast, lower RBV doses (0.1 and 1 *μ*M) did not affect viability of BV-2 cells (Figures 1(c), 1(d), and 1(f)). The percentage of late apoptotic and necrotic cells did not vary significantly among the treatments (Figure 1(f)).

### 3.2. Ribavirin Reorganizes Cytoskeleton of Activated BV-2 Cells

**Figure 2:**
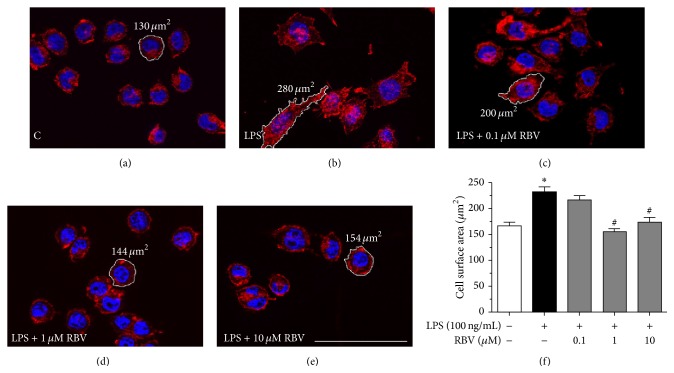
Morphology of BV-2 cells upon 24 h of LPS stimulation and RBV treatment. Phalloidin/Hoechst fluorescent staining (red/blue) of control (a), LPS (b), and LPS stimulated BV-2 cells treated with 0.1 (c), 1 (d), and 10 *μ*M RBV (e). (f) Cell surface area was measured using AxioVision Rel. 4.6 software, in five areas (138 × 104 *μ*m^2^) per each coverslip (*n* = 3) per experimental group in three independent experiments. Bars represent mean surface areas (±SEM) obtained from data presented in (a)–(e). ^*∗*^ *P* < 0.05 versus control group, ^#^ *P* < 0.05 versus LPS stimulated group. Scale bar (a)–(e): 50 *μ*m.

BV-2 cells (Figure 2(a)) stimulated with LPS developed typical morphology of activated microglia, reflected in an increase in the cell surface area and formation of multiple membrane protrusions (Figures 2(b) and 2(f)). After the RBV treatment (1 *μ*M and 10 *μ*M) cells reverted to the morphology they had before LPS stimulation (Figure 2(a)), developing round cell body with decreased cell surface area (Figures 2(d)–2(f)). Ribavirin at the lowest dose (0.1 *μ*M) failed to induce morphological changes in activated BV-2 cells (Figures 2(c) and 2(f)).

### 3.3. Ribavirin Does Not Affect Production of TNF-*α* in Activated BV-2 Cells

Morphological activation of microglia after LPS stimulation was accompanied with functional activation manifested through prominent release of proinflammatory cytokine TNF-*α* (Table 1). Nevertheless, ribavirin treatment did not affect production of this cytokine in any of the applied dosages (Table 1).

### 3.4. Low-Dose RBV Treatment Reduces LPS Induced NO Release by BV-2 Cells

**Figure 3:**
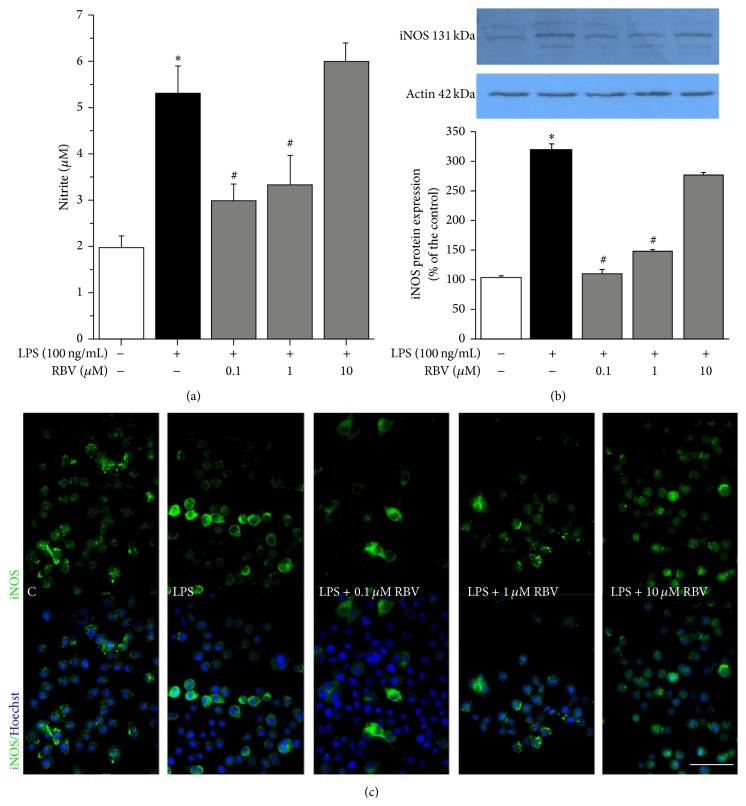
(a) Effect of RBV on LPS triggered production of NO. (b) Representative Western blot of iNOS expression. Graph shows mean iNOS protein abundance (± SEM), from *n* = 3 separate determinations, expressed relative to the abundance of *β*-actin in each lane. Significance inside the graphs (a) and (b): ^*∗*^ *P* < 0.05 versus control group, ^#^ *P* < 0.05 versus LPS stimulated group. (c) Panel of immunofluorescence labeling of BV-2 cells against iNOS (green) and counterstained with Hoechst (blue) after 24 h of stimulation with LPS and treatment with RBV. Scale bar for all pictures in panel (c): 50 *μ*m.

Potency of low-dose RBV to suppress LPS induced NO release was evaluated by measuring accumulation of nitrites in the culture medium. Surprisingly, 10 *μ*M RBV failed to induce either inhibitory or stimulatory effect on NO production, while lower RBV doses (0.1 and 1 *μ*M) decreased NO levels to about one-half of the level induced by LPS (Figure 3(a)). Since NO is the product of iNOS catalytic activity, the next step was to assess expression level of iNOS by Western blot and immunofluorescence labeling (Figures 3(b) and 3(c)). LPS induced 3-fold increase in the iNOS protein abundance, whereas RBV, at dosages 0.1 and 1 *μ*M, decreased expression of iNOS to a level comparable with the untreated cells. Ribavirin at the highest dosage (10 *μ*M) remained without effect (Figure 3(b)).

Expression of iNOS was evaluated by immunofluorescence labeling in LPS-activated BV-2 microglia (Figure 3(c)). In accordance with the Western blot analysis data, expression of iNOS was suppressed by 0.1 and 1 *μ*M RBV, while 10 *μ*M RBV was ineffective.

## 4. Discussion

It is well known that the challenge of cultured microglia with inflammatory stimuli, such as LPS, induces both activation and apoptosis of the cells [23]. The treatment-induced cell loss may represent a self-regulatory mechanism of microglial activation [24] as the cell loss was found to be involved in the resolution of inflammation [23]. Indeed, in our study LPS induced apoptosis in about 10% of BV-2 cells in culture, whereas 10 *μ*M RBV increased the number of apoptotic cells for additional 10%. The same RBV dosage has been already shown to induce comparable level of cell death in macrophages [25] and in primary microglia [21]. Since we found no evidence of significant apoptosis or necrosis with low-dose RBV treatments (0.1 and 1 *μ*M), the aim of present study was to explore if the low RBV dosages have ability to attenuate the signs of inflammatory activation of microglia* in vitro*.

The results clearly show that the low-dose RBV treatments do not affect TNF-*α* production but suppress production of NO by interfering with the induction of inducible form of NO synthase (iNOS). At the same time, low-dose RBV treatments (1 *µ*M) were effective in reverting BV-2 cell morphology to their nonactivated state, that is, the cell morphology observed in control, nonstimulated culture. These findings suggest that lower dosages of RBV, which do not induce cell death, may be equally effective in reducing the signs of microglial activation and may help explain previously published* in vivo* data, showing efficiency of RBV treatment to attenuate neuroinflammation in EAE and traumatic brain injury [16, 26].

Ribavirin efficiently reverted LPS-activated BV-2 cells into their quiescent morphology, by inducing the actin cytoskeleton rearrangement and decrease in mean cell body area. Our recent study demonstrated that efficiency of 10 *μ*M RBV to revert LPS induced alterations in cell morphology [21] was probably due to its ability to reduce intracellular GTP pool beyond the level necessary for proper organization of the cytoskeleton network [27, 28]. Present study has shown that RBV at ten times lower dose was comparably efficient to induce similar cytoskeleton reorganization without affecting BV-2 cells viability.

TNF-*α* is one of the master cytokines produced by activated microglia during inflammatory states* in vivo* [29, 30]. Results of our study have clearly demonstrated that RBV, in full range of applied dosages, did not affect production of the cytokine by LPS-activated BV-2 cells. Some previously published studies reported that RBV decreased TNF-*α* release in some cell types, including macrophages and dendritic cells [13, 14]. Therefore, results of previous and the present study suggest that modulatory effect of RBV on TNF-*α* release is cell type specific and depends on the stimuli [13] and RBV dosage [31].

Nitric oxide is important regulatory molecule in the CNS, in both physiological and pathological conditions. It may exert both neuroprotective and neurotoxic effects depending on its overall concentration [32]. In general, overproduction of NO by activated microglial cell is one of the hallmarks of neuroinflammation [33, 34] and the response is associated with the progression of several neurodegenerative diseases [32, 35]. After the inflammatory challenge, level of NO increases severalfold and for a prolonged period of time, due to the induction of inducible form of NOS, which may lead to nitrosative stress and cell death. Therefore, agents with the ability to interfere with iNOS expression may be beneficial in the treatment of conditions associated with the overproduction of NO, including septic shock, inflammation, and neurodegenerative diseases [32, 36]. In our* in vitro* model, low-dose RBV treatment reduced iNOS expression and production of NO, as it was previously reported [37–39]. The effect of RBV was probably due to the inhibition of the catalytic action of IMPDH, which induces a reduction of the intracellular pool of guanosine-based nucleotides and consequently decreases level of tetrahydrobiopterin, which is cofactor required for the iNOS activity [38, 40]. It has been recently shown that RBV reduces rate of nucleocytoplasmic transport of iNOS mRNA in IMPDH independent pathway [41], indicating that RBV modulates iNOS expression through multiple ways. Nevertheless, the effect of RBV seems to be dose dependent, as RBV at dose of 10 *μ*M failed to produce any effect on iNOS expression and NO release.

Here we employed pretreatment with RBV and showed its potential preventative ability. However, previously we demonstrated that RBV is efficient in terminating neuroinflammation* in vivo* [42, 43] and our earlier* in vitro* research also showed the potential of this drug to modulate activated microglia even when applied simultaneously with LPS [21]. Altogether, RBV is a drug with potential preventative and therapeutic property in neuroinflammation.

## 5. Conclusion

Given the fact that overproduction of NO by activated microglia during the neuroinflammation leads to the development of reactive nitrogen species, such as peroxynitrite, nitrogen dioxide, which exert devastating effects on the neuronal cells (reviewed in [32]), obtained results strongly suggest that low-dose RBV treatment may be neuroprotective, due to low toxicity and high efficiency in NO suppression. Attenuation of nitrosative stress through the modulation of iNOS could be beneficial if applied early in neuroinflammatory states associated with demyelinating diseases [44, 45], such as multiple sclerosis.

## Figures and Tables

**Table 1:** Release of TNF-*α* from activated BV-2 cells treated with RBV.

Groups	C	LPS	LPS + 0.1 *μ*M RBV	LPS + 1 *μ*M RBV	LPS + 10 *μ*M RBV

TNF-*α* (pg/mL)	50 ± 23	2110 ± 95^*∗*^	2114 ± 59	2361 ± 84	2157 ± 175

^*∗*^ *P* < 0.05 versus control group (C).
